# LESSONS LEARNED DEVELOPING A NUTRITIONAL ADHERENCE INTERVENTION FOR DEMENTIA PREVENTION: A QUALITATIVE ANALYSIS

**DOI:** 10.1093/geroni/igad104.1306

**Published:** 2023-12-21

**Authors:** Julia Sheffler, Juhi Patel, Dimitris Kiosses, Sylvie Naar

**Affiliations:** Florida State University College of Medicine, Tallahassee, Florida, United States; Florida State University, Tallahassee, Florida, United States; Weill Cornell Medicine, New York City, New York, United States; Florida State University, Tallahassee, Florida, United States

## Abstract

Lifestyle interventions show considerable promise in delaying the onset and reducing risk for Alzheimer’s disease and related dementias (ADRD). Unfortunately, scalable interventions that address issues of long-term adherence have not been adequately developed or disseminated. Especially important for this effort is iterative evaluation and adaptation of interventions using participant feedback throughout the development process. Using the NIH ORBIT model for behavioral intervention development, our team has established the proof-of-concept (N=9) and pilot tested (N=58) a Mediterranean ketogenic nutrition (MKN) adherence program for older adults at risk for ADRD. This program incorporates motivational interviewing strategies and cognitive behavioral skills training to enhance uptake and long-term adherence to MKN. For each of these pilots, participants completed exit interviews at the conclusion of the 6-week, group intervention and 3-months post-intervention. Qualitative and quantitative data on the acceptability of the program, as well as detailed participant feedback about their experience and recommendations for improvements were collected. While quantitative data demonstrate high acceptability, thematic analysis of qualitative interviews demonstrate a range of recommendations for enhancing and refining important components of the intervention that will be necessary for future scaling and dissemination. We discuss important themes, future directions of this work, and implications for lifestyle intervention development more broadly.

